# NCAPD2 promotes the progression of lung adenocarcinoma through an AKT/MDM2/E2F1 positive feedback loop

**DOI:** 10.1080/15384047.2025.2589678

**Published:** 2025-11-30

**Authors:** Yun Wu, Xiaoqin Li, Yuchen Lin, Yan Chen, Ning Xin, Da Hong, Junmin Wei, Hongru Li, Tailin Guo, Fan Lin, Yusheng Chen, Ying Lin

**Affiliations:** a Fujian Provincial Center for Geriatrics, Fuzhou University Affiliated Provincial Hospital, Fuzhou, China; b Shengli Clinical Medical College of Fujian Medical University, Fuzhou, China; c Department of General Practice Medicine, Fujian Provincial Hospital, Fuzhou, China; d Department of Pulmonary and Critical Care Medicine, Fuzhou University Affiliated Provincial Hospital, Fuzhou, China; e The School of Nursing, Fujian Medical University, Fuzhou, China; f Department of Pathology, Fuzhou University Affiliated Provincial Hospital, Fuzhou, China

**Keywords:** Lung adenocarcinoma, NCAPD2, PI3K/AKT/MDM2 pathway, E2F1, positive feedback loop

## Abstract

**Introduction:**

Lung adenocarcinoma (LUAD) is one of the leading causes of cancer-related deaths worldwide. While NCAPD2 has been implicated in promoting tumorigenesis across various cancer types, its specific role in LUAD remains underexplored. This study aims to elucidate the molecular mechanisms by which NCAPD2 contributes to LUAD progression, with a focus on its involvement in the AKTMDM2/E2F1 positive feedback loop.

**Materials and methods:**

NCAPD2 expression in LUAD and normal tissues was analyzed using Western blotting and immunohistochemistry (IHC). Functional assays, including colony formation, wound healing, Transwell assays, and in vivo mouse models were conducted to evaluate the impact of NCAPD2 on LUAD cell proliferation, invasion, and metastasis. RNA sequencing and protein interaction experiments were used to investigate the role of NCAPD2 in the PI3K/AKT/MDM2 pathway and its interaction with E2F1.

**Results:**

This study first identified that NCAPD2 expression is significantly upregulated in LUAD tissues, particularly in higher pathological stages. NCAPD2 overexpression promoted LUAD cell proliferation and metastasis, while its knockdown inhibited tumor growth and invasion. Mechanistically, NCAPD2 activated the PI3K/Akt pathway, facilitating the interaction between MDM2 and E2F1, reducing E2F1 ubiquitination, and increasing its expression. Furthermore, E2F1 enhanced NCAPD2 transcription, forming a positive feedback loop that drives LUAD progression.

**Conclusion:**

This study reveals a novel role of NCAPD2 in promoting LUAD progression through the AKT/MDM2/E2F1 positive feedback loop. These findings provide new insights into the molecular pathogenesis of LUAD and suggest NCAPD2 as a potential therapeutic target for improving patient outcomes.

## Graphical abstract

**Figure:**
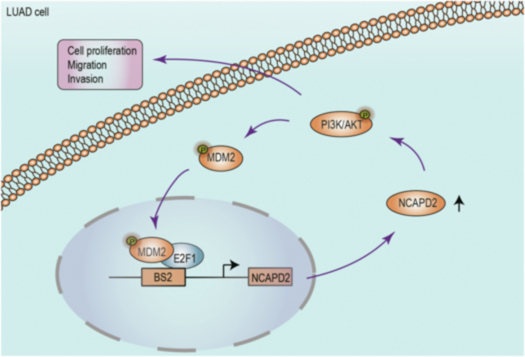

## Introduction

Lung cancer remains one of the deadliest malignancies worldwide, significantly affecting public health. Approximately 2.21 million new cases of lung cancer were reported globally in 2020.^1^ Among them, non-small cell lung cancer (NSCLC) constitutes the most prevalent type, accounting for 85% of all lung cancer cases. Adenocarcinoma represents the majority of NSCLC cases, approximately 40%–70%. Despite advances in early detection and treatment, the 5-y survival rate of NSCLC remains dismal, particularly for patients diagnosed at advanced stages.^2^^,^^3^

Condensin complexes are essential for chromosome condensation and segregation, and dysregulation of their subunits has been increasingly linked to tumorigenesis. Among these subunits, NCAPD2, a component of condensin I, has recently been implicated in several cancers. For example, Zhang et al. first reported its association with triple-negative breast cancer,^4^ and subsequent studies revealed that NCAPD2 promotes colorectal cancer progression through the Ca^2+^/CAMKK2/AMPK/mTORC1 pathway and the PARP-1/SIRT1 axis.^5^

However, the role of NCAPD2 in lung adenocarcinoma (LUAD) remains largely unexplored. Preliminary analyses indicated that NCAPD2 expression is significantly upregulated in LUAD compared with adjacent normal tissues. Therefore, this study specifically focused on NCAPD2 and investigated the molecular mechanisms by which it contributes to LUAD progression, with particular attention to its involvement in the AKT/MDM2/E2F1 signaling axis.

## Results

### Expression and prognostic significance of condensin subunits in LUAD

We analyzed the expression of condensin protein complex subunits in various cancers using the Tumor IMmune Estimation Resource (TIMER) database and determined their widespread overexpression. In LUAD, SMC2, SMC4, NCAPG, NCAPH, NCAPD2, NCAPG2, and NCAPD3 were significantly upregulated compared to adjacent tissues, while NCAPH2 showed no difference (Figure S1). Protein expression analysis using UALCAN (University of Alabama at Birmingham Cancer data analysis portal) revealed significant differences in all subunits except NCAPD3 (Figure S2). High expression of SMC2, SMC4, NCAPG, NCAPH, NCAPD2, and NCAPG2 was correlated with shorter survival in patients, emphasizing their prognostic significance (Figure S3). Furthermore, we conducted univariate and multivariate Cox regression analyses to assess the correlation between these genes and clinical factors, such as age, gender, clinical stage, T stage, N stage, and M stage, identifying SMC2, NCAPG, NCAPH, NCAPD2, and NCAPG2 as independent risk factors for lung adenocarcinoma (Table S3).

**Figure 1.**
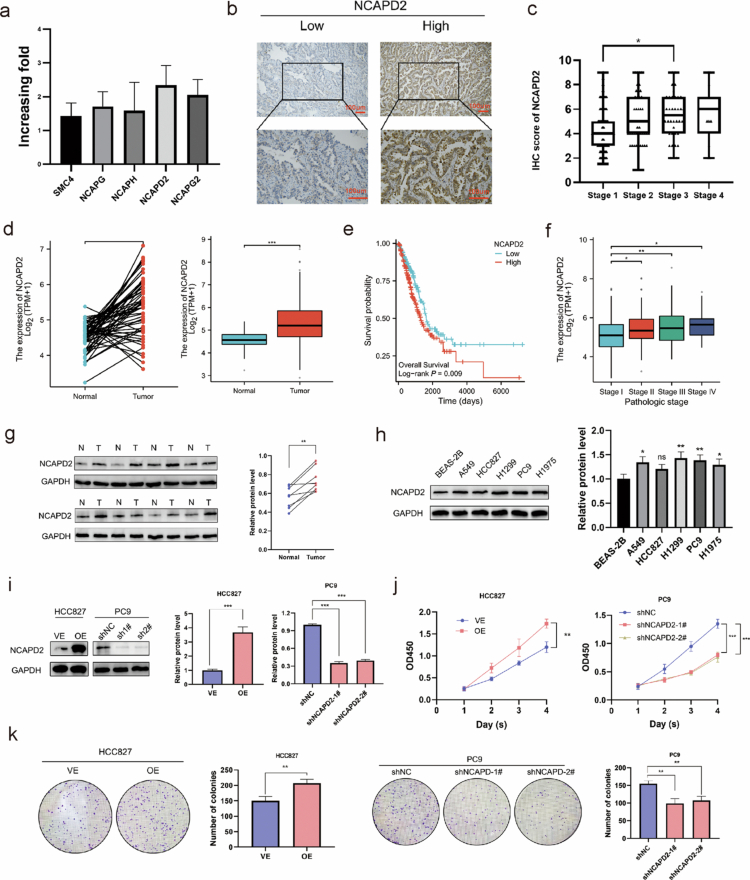
Overexpression of NCAPD2 in lung adenocarcinoma and its promotion of proliferation. a. NCAPD2 expression was the most significant difference in expression between lung adenocarcinoma tissues and adjacent tissues. b. Representative immunohistochemical staining images of NCAPD2. c. Correlation between NCAPD2 expression and pathological stage. d. Elevated expression of NCAPD2 in lung adenocarcinoma patients compared to normal lung tissues (TCGA database). e. High NCAPD2 expression is correlated with poor survival. f. NCAPD2 expression increases with advancing clinical stage. g. Western blot analysis showing increased NCAPD2 expression in LUAD tissues compared to adjacent normal lung tissues from 8 paired samples. h. Increased NCAPD2 expression in lung adenocarcinoma cell lines (A549, PC9, HCC827, H1975, and H1299) compared to alveolar epithelial cells (BEAS-2B) (western blotting). i. Significant knockdown of NCAPD2 with shNCAPD2-1# and shNCAPD2-2# at the protein level, while OE-NCAPD2 exhibits higher protein levels than those in the control group (western blotting). j and k. NCAPD2 overexpression promotes proliferation in HCC827 cells (CCK-8 and colony formation assays), while NCAPD2 knockdown inhibits proliferation in PC9 cells. *p < 0.05, **p < 0.01, and ***p < 0.001. VE: empty vector control, OE: overexpression, sh: short hairpin, TPM: transcripts per million.

Next, we quantified the relative mRNA expression levels of SMC2, NCAPG, NCAPH, NCAPD2, and NCAPG2 in 8 pairs of fresh LUAD and adjacent normal tissues using quantitative real-time polymerase chain reaction (qRT-PCR). We observed the most significant difference in expression between LUAD tissues and adjacent tissues in the case of NCAPD2 (Figure 1a).

Subsequently, we collected 207 LUAD paraffin-embedded tissue sections with detailed clinical and pathological data. Immunohistochemical staining analyzed the expression of NCAPD2. Among the cases, 107 were stage I, 49 were stage II, 38 were stage III, and 13 were stage IV. IHC showed NCAPD2 mainly in the cytoplasm (Figure 1b). Immunohistochemical scoring based on staining intensity was performed. The expression levels of NCAPD2 generally increase with the progression of pathological stage (Figure 1c).

In summary, these analyses highlight NCAPD2 as the most significantly upregulated condensin subunit in LUAD and strongly associated with advanced pathological stage and poor prognosis. This evidence supported our decision to focus subsequent mechanistic studies on NCAPD2.

### Overexpression of NCAPD2 in LUAD

The mRNA expression level of NCAPD2 was significantly elevated in LUAD patients from The Cancer Genome Atlas (TCGA) database compared with normal lung tissues (Figure 1d). Moreover, higher NCAPD2 expression was associated with poor overall survival (Figure 1e) and showed a progressive increase with advancing clinical stage (Figure 1f). In addition, Western blot analysis of eight paired LUAD and adjacent normal lung tissues revealed consistently higher NCAPD2 protein levels in LUAD samples (Figure 1g).

Together, these data confirm that NCAPD2 is consistently upregulated at both mRNA and protein levels in LUAD, correlates with disease stage, and predicts poor patient outcomes. These findings further validate the clinical relevance and oncogenic potential.

### NCAPD2 promotes the proliferation of LUAD cells

Western blot analysis revealed that NCAPD2 expression was higher in NSCLC cell lines (A549, PC9, HCC827, H1975, and H1299) than in normal alveolar epithelial cells (BEAS-2B) (Figure 1h). Based on the relatively high NCAPD2 level in PC9 cells and the low level in HCC827 cells—both harboring EGFR mutations—these two lines were selected for subsequent functional experiments. Specifically, PC9 cells were transfected with shNCAPD2-1# and shNCAPD2-2# for knockdown, whereas HCC827 cells were transfected with an NCAPD2 overexpression plasmid (OE-NCAPD2). Western blotting confirmed efficient knockdown by shNCAPD2-1# and shNCAPD2-2# and marked overexpression in OE-NCAPD2 cells (Figure 1i). Functional assays, including Cell Counting Kit-8 (CCK-8) and colony formation assays, showed that NCAPD2 overexpression promoted, while NCAPD2 knockdown inhibited, LUAD cell proliferation (Figure 1j, k). To further validate these findings, reciprocal experiments were performed—NCAPD2 overexpression in PC9 cells and knockdown in HCC827 cells—with consistent results presented in the supplementary materials (Figure S4a–c).

In summary, functional assays consistently demonstrated that NCAPD2 enhances LUAD cell proliferation. Knockdown of NCAPD2 reduced cell growth, while overexpression of NCAPD2 significantly increased it.

### NCAPD2 facilitates cell cycle progression in LUAD cells

**Figure 2.**
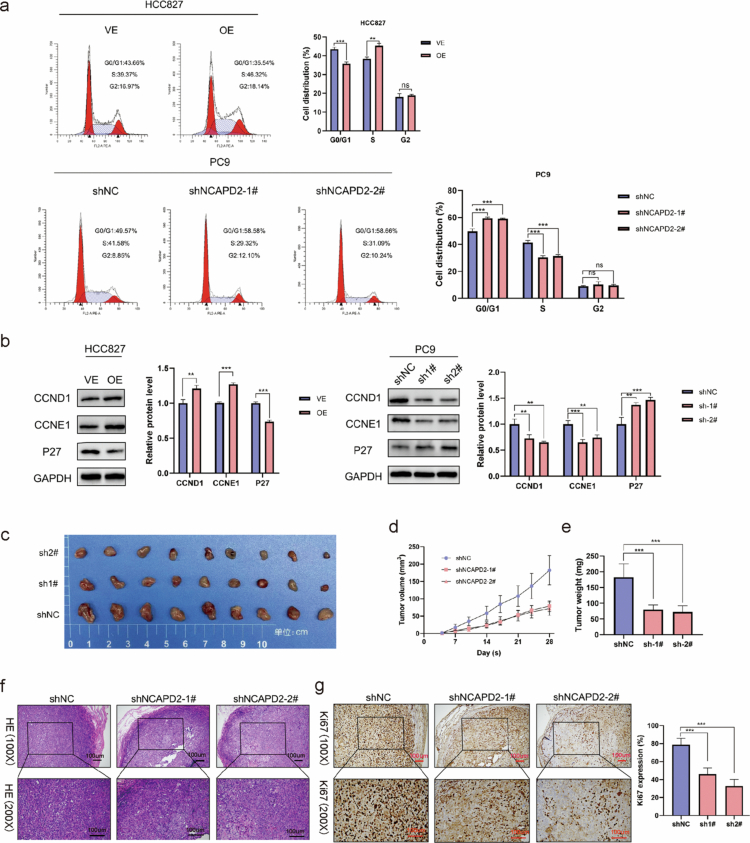
Effects of NCAPD2 on cell cycle progression and in vivo tumor growth in lung adenocarcinoma. a. NCAPD2 alters the cell cycle distribution of HCC827 and PC9 cells. b. Expression of cell cycle-related markers (CCND1, CCNE1, P27) in PC9 and HCC827 cells. c. Photographs of xenograft tumors. d-e. The volume and mass of tumors formed by the NCAPD2 knockdown cell lines were significantly smaller compared to the control group. f and g. H&E staining and immunohistochemical staining of Ki-67 in xenograft tumors. *p < 0.05, **p < 0.01, and ***p < 0.001. (H&E): hematoxylin and eosin.

The impact of NCAPD2 on the cell cycle was assessed using flow cytometry. The overexpression of NCAPD2 decreased the G0/G1 phase and increased the S phase in HCC827 cells, while knockdown of NCAPD2 had the opposite effect in PC9 cells (Figure 2a). Western blot analysis of cell cycle-related proteins (Figure 2b) showed increased expression of CCND1 and CCNE1 with NCAPD2 overexpression, while their expression decreased with NCAPD2 knockdown. P27, an inhibitor of cell cycle progression, displayed reduced expression with NCAPD2 overexpression and increased expression with NCAPD2 knockdown.

### NCAPD2 promotes the proliferation of LUAD in vivo

To investigate the impact of NCAPD2 on tumor proliferation in vivo, subcutaneous tumor formation experiments were conducted in nude mice using control PC9 cell lines and stable NCAPD2 knockdown PC9 cells. The tumor size was measured approximately twice per week for 28 d. Tumors formed by the NCAPD2 knockdown cell lines were significantly smaller in volume and mass compared to the control group (Figure 2c–e). The maximum tumor diameter measured was 9.7 mm, and the tumor volume was 211 mm³. Additionally, H&E and Ki67 staining were performed on sections of the fixed tumor tissue, revealing a lower Ki67-positive rate in tumors formed by NCAPD2 knockdown PC9 cells than in the control group (Figure 2f, g).

In summary, in vivo xenograft assays confirmed that silencing NCAPD2 markedly reduced tumor growth and proliferation, which was consistent with the in vitro findings. These results establish NCAPD2 as a critical driver of LUAD tumorigenicity.

### NCAPD2 promotes LUAD migration, invasion, EMT, and metastasis

**Figure 3.**
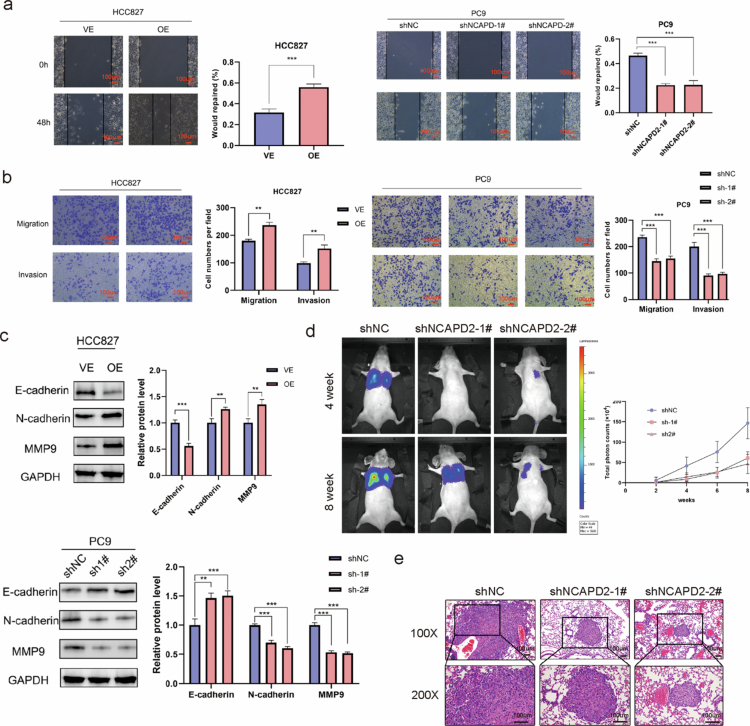
NCAPD2 promotes migration, invasion, and epithelial‒mesenchymal transition (EMT) in lung adenocarcinoma cells. a. NCAPD2 overexpression enhances HCC827 cell migration (wound healing assay), while NCAPD2 knockdown weakens PC9 cell migration. b. NCAPD2 overexpression increases HCC827 cell migration and invasion (transwell assay), while NCAPD2 knockdown decreases migration and invasion in PC9 cells. c. Expression of EMT-related markers (E-cadherin, N-cadherin, and MMP9) in PC9 and HCC827 cells. d. Assessment of invasive and metastatic capacities via small animal in vivo imaging following NCAPD2 knockdown. e. Representative H&E images of lung metastases in the mouse model. *p < 0.05, **p < 0.01, and ***p < 0.001.

The impact of NCAPD2 on LUAD cell migration and invasion was assessed using wound healing and Transwell assays. NCAPD2 overexpression promoted the cell migration and invasion of HCC827 cells, while in PC9 cells NCAPD2 knockdown inhibited these processes (Figure 3a, b). To further validate these findings, reciprocal experiments were performed, including wound-healing and Transwell assays with PC9 cells overexpressing NCAPD2 and HCC827 cells with NCAPD2 knockdown. Consistent results were obtained—NCAPD2 overexpression enhanced, while its knockdown reduced, migratory and invasive capacities (Figure S4d,e). These findings indicate the role of NCAPD2 in regulating LUAD EGFR-mutant cell's migration and invasion. In addition to PC9 and HCC827 cell lines, we further validated the role of NCAPD2 in an EGFR wild-type LUAD cell line A549. Consistent with our previous findings, the overexpression of NCAPD2 in A549 cells significantly promoted cell proliferation, migration, and invasion, while knockdown of NCAPD2 resulted in the opposite effects (Figure S4f–h). These results suggest that the oncogenic role of NCAPD2 in promoting LUAD progression may not be limited to EGFR-mutant cell lines, supporting its broader relevance in LUAD.

To investigate the effect of NCAPD2 on epithelial‒mesenchymal transition (EMT), we examined the expression of E-cadherin, N-cadherin, and MMP9. NCAPD2 overexpression led to decreased E-cadherin expression, increased N-cadherin expression, and upregulation of MMP9. Conversely, NCAPD2 knockdown resulted in increased E-cadherin expression, decreased N-cadherin expression, and downregulation of MMP9 (Figure 3c). These findings suggest the involvement of NCAPD2 in regulating EMT in LUAD cells.

The impact of NCAPD2 on the invasive and metastatic capabilities of lung adenocarcinoma (LUAD) cells in vivo were investigated through a tail vein injection model. Luc-PC9 stable control cells and NCAPD2 knockdown cells, both labeled with luciferase, were injected, and tumor growth was monitored biweekly using live animal imaging. The results revealed a significant reduction in the metastasis of lung cancer cells upon NCAPD2 knockdown, evidenced by a marked decrease in the total photon count in lung tumors (Figure 3d). At the 8th week, the mice were euthanized, and the lung tissues were fixed in 10% neutral formalin. Histological examination of lung sections stained with hematoxylin and eosin (H&E), demonstrated diverse tumor cell morphologies, nuclear pleomorphism, and pathological mitotic figures in metastatic foci (Figure 3e).

In summary, both in vitro and in vivo data demonstrated that NCAPD2 promotes LUAD migration, invasion, and metastasis, at least in part through the induction of EMT. These findings establish NCAPD2 as a potent driver of LUAD metastatic progression.

### NCAPD2 promotes LUAD progression through the PI3K/AKT signaling pathway

**Figure 4.**
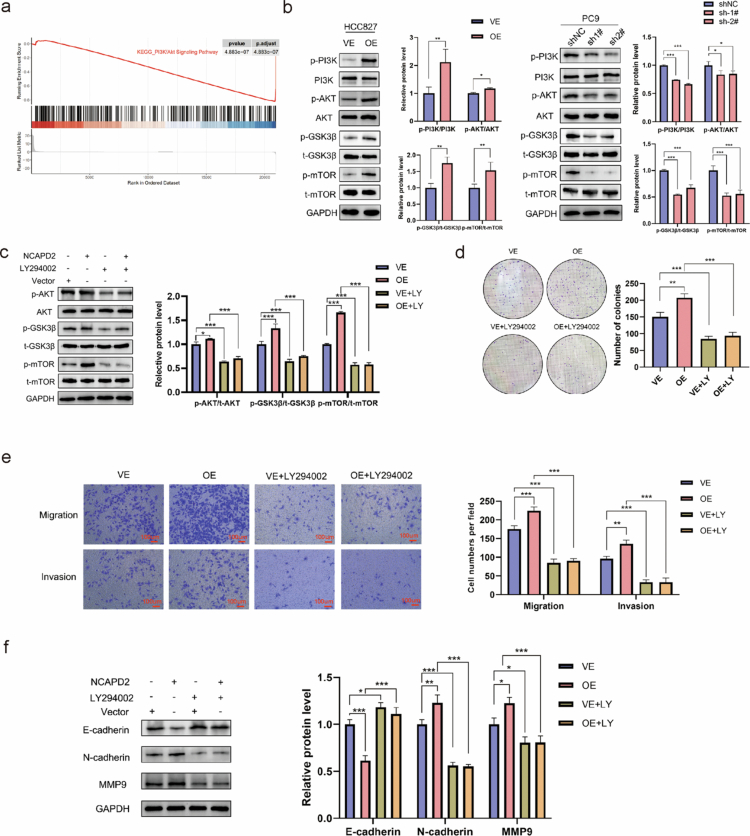
NCAPD2 promotes lung adenocarcinoma progression via the PI3K/AKT signaling pathway. a. GSEA analysis showed downregulation of PI3K/AKT pathway in NCAPD2 knockdown cells. b. NCAPD2 promotes lung adenocarcinoma via PI3K/AKT phosphorylation. c. LY294002 reversed the PI3K/AKT activation caused by NCAPD2 overexpression. d. LY294002 eliminates the increased proliferation induced by NCAPD2 overexpression. e. LY294002 restored heightened invasion in NCAPD2-overexpressing cells. f. LY294002 abolishes the altered expression of E-cadherin, N-cadherin, and MMP9 in NCAPD2-overexpressing cells. LY294002: a PI3K protein kinase inhibitor. *p < 0.05, **p < 0.01, and ***p < 0.001.

RNA sequencing was performed on NCAPD2 control and knockdown treated PC9 cells to identify the NCAPD2-enhanced and inhibited transcriptome. GSEA enrichment analysis highlighted the involvement of the PI3K/AKT signaling pathway (Figure 4a). Western blotting showed higher levels of p-PI3K, p-AKT, p-GSK3β, and p-mTOR in NCAPD2-overexpressing HCC827 cells, while PC9 cells with NCAPD2 knockdown exhibited reduced levels of p-PI3K, p-AKT, p-GSK3β, and p-mTOR (Figure 4b). These phosphorylations indicate activation of the PI3K/AKT signaling pathway, suggesting that NCAPD2 may contribute to LUAD progression by enhancing this pathway.

To confirm this, a rescue assay was conducted using the PI3K inhibitor LY294002 (LY294002). Western blotting showed that LY294002 could reverse the activation of the PI3K/AKT pathway caused by the overexpression of NCAPD2 (Figure 4c). LY294002 also reversed the enhanced proliferation, migration, and invasion induced by NCAPD2 overexpression (Figure 4d, e). Additionally, the expression changes in E-cadherin, N-cadherin, and MMP9 caused by NCAPD2 overexpression were abolished by LY294002 (Figure 4f).

In summary, transcriptomic profiling, Western blotting, and pharmacological inhibition collectively demonstrated that NCAPD2 promotes LUAD progression by activating the PI3K/AKT signaling pathway, which subsequently regulates proliferation, migration, invasion, and EMT.

### Transcription factor E2F1 positively regulates the expression of NCAPD2 in LUAD

**Figure 5.**
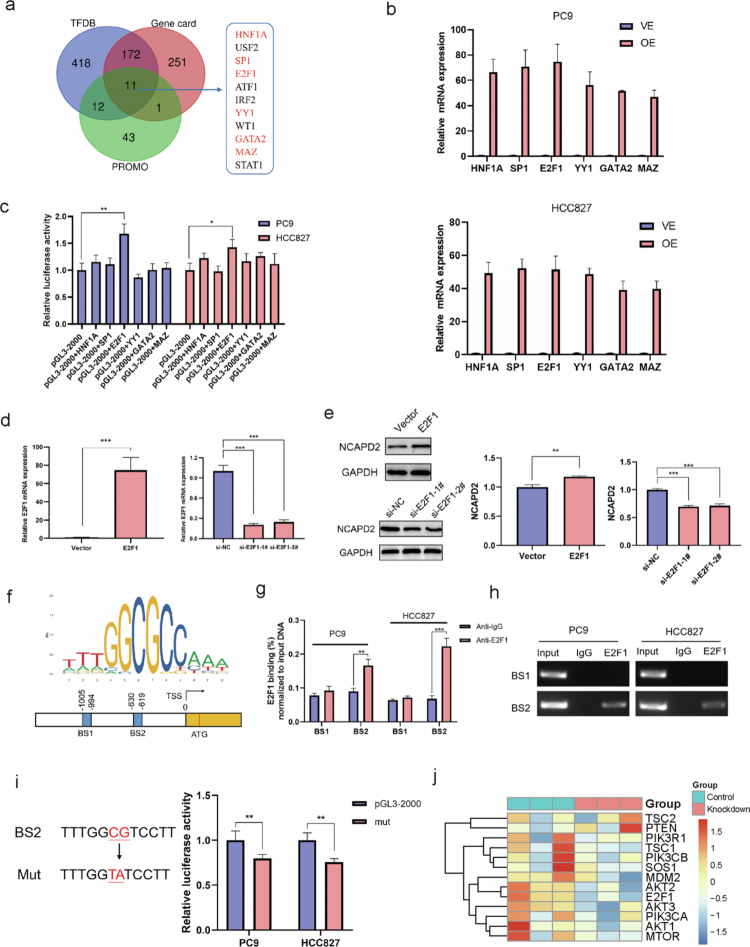
The transcription factor E2F1 positively regulates NCAPD2 expression in lung adenocarcinoma. a. HNF1A, SP1, E2F1, YY1, GATA2, and MAZ were identified as potential regulators of NCAPD2 based on binding site analysis. b. Overexpression efficiencies of HNF1A, SP1, E2F1, YY1, GATA2, and MAZ in HCC827 and PC9 cells, as verified by qRT-PCR. c. E2F1 exhibited significantly higher transcriptional activity compared to other transcription factors, including HNF1A, SP1, YY1, GATA2, and MAZ, when co-transfected with the pGL3-2000 luciferase reporter plasmid into PC9 and HCC827 cells. d. E2F1 knockdown and overexpression resulted in significant changes in mRNA expression level. e. Overexpression of E2F1 increased NCAPD2 expression, while E2F1 knockdown had the opposite effect. f. JASPAR predicted two potential E2F1 binding sites in the NCAPD2 promoter region. g. ChIP assay confirmed that E2F1 binds to the BS2 site but not the BS1 site. h. PCR products from ChIP were visualized by gel electrophoresis, supporting specific binding of E2F1 to the BS2 site. i. DR assay showed reduced luciferase activity with the BS2 mutation site. j. The heatmap depicts the differential expression of key genes in the PI3K/AKT pathway and E2F1 across PC9 cell lines, comparing the control group and NCAPD2 knockdown group. *p < 0.05, **p < 0.01, and ***p < 0.001. DR: double luciferase reporter, ChIP: chromatin immunoprecipitation.

Transcription factor regulation is crucial in tumor progression. We screened potential factors influencing NCAPD2 expression using the Transcription Factor Database (TFDB), GeneCards database, and PROMO prediction tool. The analysis revealed 11 potential factors (HNF1A, USF2, SP1, E2F1, ATF1, IRF2, YY1, WT1, GATA2, MAZ, and STAT1). Further screening with the nonredundant transcription factor binding motif database (JASPAR) allowed us to narrow down six factors (HNF1A, SP1, E2F1, YY1, GATA2, and MAZ) with binding scores > 8 on the NCAPD2 promoter (Figure 5a).

The overexpression efficiencies of HNF1A, SP1, E2F1, YY1, GATA2, and MAZ in HCC827 and PC9 cells were verified by qRT-PCR (Figure 5b). Cotransfection of these transcription factors with the pGL3-2000 luciferase reporter plasmid into PC9 and HCC827 cells, followed by luciferase reporter assays normalized to firefly/Renilla ratios, demonstrated that E2F1 had higher transcriptional activity than others (Figure 5c). We subsequently focused on E2F1 and transfected overexpression and knockdown vectors into HCC827 and PC9 cells. E2F1 knockdown and overexpression significantly affected E2F1 mRNA expression (Figure 5d), and the direction of NCAPD2 expression was consistent with the changes in E2F1 (Figure 5e). These results support E2F1's positive regulation of NCAPD2.

JASPAR prediction identified two E2F1 binding sites (BS1: −1005 nt to −994 nt; BS2: −630 nt to −619 nt) on the NCAPD2 promoter (Figure 5f). A chromatin immunoprecipitation (ChIP) assay with an E2F1 antibody showed specific binding to the BS2 site, not BS1 (Figure 5g), which was supported by gel electrophoresis (Figure 5h). This suggests direct interaction between E2F1 and the BS2 site.

To confirm this binding, we designed a plasmid with a mutated BS2 site and cotransfected it with E2F1. A luciferase reporter assay revealed reduced activity in the mutated plasmid (Figure 5i). These findings indicate that E2F1-regulated NCAPD2 transcription is enabled by specific binding to the BS2 site (−630nt to −619nt) in the NCAPD2 promoter. Additionally, we have included a heatmap showing the expression levels of PI3K, AKT, and E2F1 from our RNA sequencing data (Figure 5j), which provides a visual representation of the pathway activity and further substantiates our findings.

In summary, E2F1 directly binds to the NCAPD2 promoter (at BS2: −630 to −619 nt) and activates its transcription, thereby establishing a regulatory link between NCAPD2 and the PI3K/AKT/E2F1 axis.

### NCAPD2 regulates the progression of LUAD through the AKT/MDM2/E2F1 positive feedback loop

**Figure 6.**
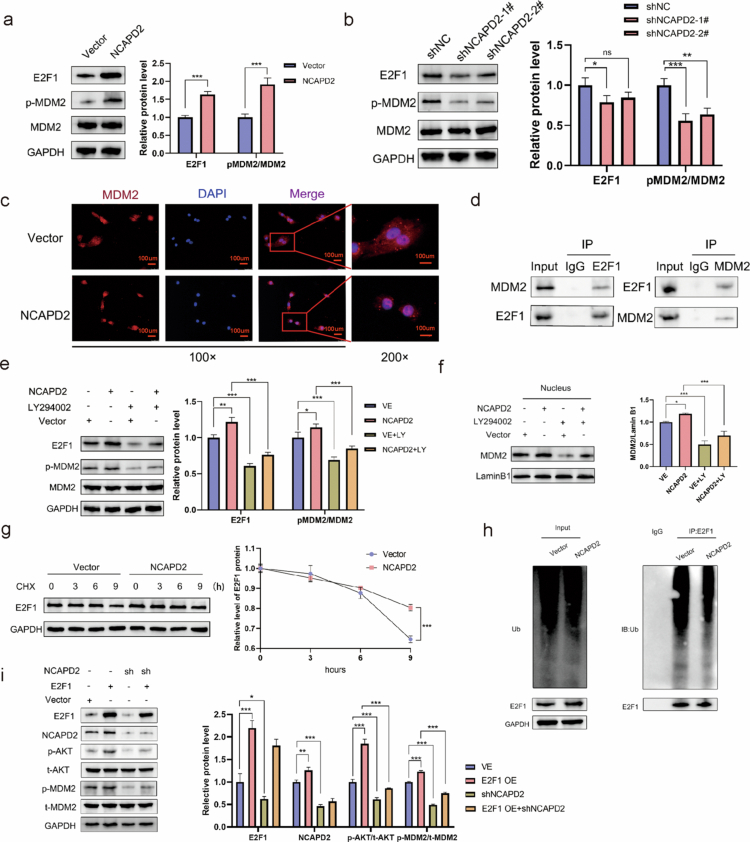
NCAPD2 regulates the progression of lung adenocarcinoma through the AKT/MDM2/E2F1 positive feedback loop. a and b. NCAPD2 overexpression upregulated E2F1 and p-MDM2 protein levels, while knockdown downregulated them. c. NCAPD2 overexpression enhanced MDM2 nuclear translocation. d. MDM2 and E2F1 were found to interact through co-immunoprecipitation. e. LY294002 treatment reversed the increased expression of E2F1 and p-MDM2 caused by NCAPD2 overexpression. f. LY294002 prevented the nuclear increase of MDM2 in NCAPD2-overexpressing cells. g. NCAPD2 overexpression extended the half-life of E2F1 in the presence of CHX. h. Immunoprecipitation assay showing reduced ubiquitination of E2F1 upon NCAPD2 overexpression. i. Overexpression of E2F1 enhanced the activation of AKT and MDM2, while knockdown of NCAPD2 significantly reduced the phosphorylation levels of both AKT and MDM2. *p < 0.05, **p < 0.01, ***p < 0.001.

AKT activation leads to the phosphorylation of multiple downstream factors, including the crucial target gene MDM2. Previous research has demonstrated that MDM2 can extend the half-life of E2F1 by inhibiting its ubiquitination, which is facilitated by its nuclear colocalization.^6^ Moreover, it has been established that E2F1 acts as a downstream effector of the PI3K/AKT pathway.^7^ To assess the impact of NCAPD2 on the protein expression levels of p-MDM2, MDM2, and E2F1, we conducted Western blotting experiments. Our findings revealed a significant increase in the protein expression levels of E2F1 and p-MDM2 upon NCAPD2 overexpression (Figure 6a), while knockdown decreased their levels (Figure 6b). Immunofluorescence experiments further confirmed the promotion of MDM2 nuclear translocation by NCAPD2 overexpression (Figure 6c), and co-immunoprecipitation (Co-IP) validated the MDM2-E2F1 interaction (Figure 6d).

To ascertain whether the changes in p-MDM2 and E2F1 induced by NCAPD2 overexpression were attributed to the activation of the PI3K/AKT pathway, Western blot analysis was performed on LY294002-treated NCAPD2-overexpressing cells. LY294002 effectively restored the heightened expression levels of E2F1 and p-MDM2 induced by NCAPD2 overexpression (Figure 6e). Additionally, treatment with LY294002 effectively abolished the increase in nuclear MDM2 observed in HCC827 cells with NCAPD2 overexpression (Figure 6f).

In subsequent experiments, we treated HCC827 cells overexpressing NCAPD2 and negative control cells with the protein synthesis inhibitor cycloheximide (CHX, 10 μg/ml) to assess the half-life of E2F1. The results demonstrated that the half-life of E2F1 was significantly prolonged in NCAPD2-overexpressing cells compared to the respective control group cells (Figure 6g), increasing from approximately 16 h in control cells to about 30 h in NCAPD2-overexpressing cells. Furthermore, ubiquitin experiments demonstrated a reduction in the ubiquitination level of E2F1 following NCAPD2 overexpression (Figure 6h). To further validate whether E2F1's regulation of the PI3K/AKT signaling pathway is mediated through NCAPD2, we conducted a rescue experiment in which E2F1 was overexpressed and NCAPD2 was subsequently knocked down. The results showed that knocking down NCAPD2 significantly suppressed the activation of p-AKT and p-MDM2, which were initially upregulated by E2F1 overexpression (Figure 6i). This finding supports the hypothesis that E2F1 activates the PI3K/AKT signaling pathway through NCAPD2, highlighting NCAPD2's crucial role in mediating E2F1's oncogenic effects in LUAD progression.

Notably, E2F1 also acts downstream of NCAPD2 through a feedback mechanism. To further verify the causal relationship within the NCAPD2/AKT/MDM2/E2F1 signaling pathway, we performed a rescue experiment and a requirement experiment. In the rescue experiment, E2F1 was overexpressed in NCAPD2 knockdown LUAD cells. Western blot analysis showed that NCAPD2 silencing markedly reduced *p*-AKT (Ser473), p-MDM2 (Ser166), and E2F1 levels, whereas E2F1 overexpression restored these signals (Figure S5a). Functional assays further demonstrated that NCAPD2 knockdown significantly inhibited cell proliferation (CCK-8 assay) as well as cell migration and invasion (Transwell assay), and these inhibitory effects were partially rescued by E2F1 overexpression (Figure S5b,c). In the requirement experiment, NCAPD2 was overexpressed in LUAD cells with or without MDM2 knockdown. Overexpression of NCAPD2 elevated p-AKT, *p-M*DM2, and E2F1 expression and markedly enhanced proliferation, migration, and invasion, whereas MDM2 silencing abolished these effects while p-AKT remained elevated (Figure S5d–f). Collectively, these results indicate that E2F1 is sufficient to compensate for NCAPD2 loss, whereas MDM2 is required for NCAPD2-mediated activation of E2F1 and its downstream oncogenic phenotypes, confirming the causal order of NCAPD2/AKT/MDM2/E2F1 in LUAD progression.

**Figure 7.**
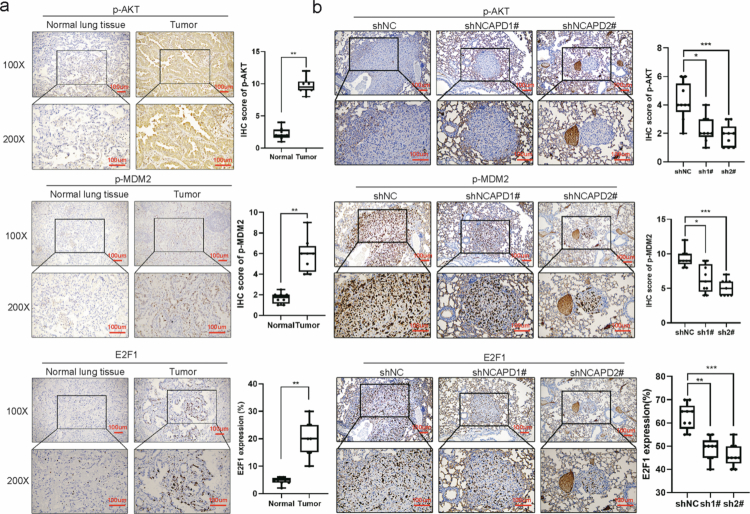
Immunohistochemical analysis of AKT/MDM2/E2F1 pathway markers in human LUAD tissues and adjacent normal tissues, as well as in BALB/c nude mouse lung metastasis models. a. The expression levels of p-AKT, p-MDM2, and E2F1 are higher in human lung cancer tissues compared to adjacent normal lung tissues. b. In the BALB/c nude mouse lung metastasis model, the expression of these markers is elevated in the control group (shNC) compared to the NCAPD2 knockdown groups (sh-1# and sh-2#), suggesting the involvement of NCAPD2 in activating the AKT/MDM2/E2F1 signaling pathway. *p < 0.05, **p < 0.01, ***p < 0.001.

In order to further understand the impact of NCAPD2 on LUAD progression, we performed IHC analysis on both human lung cancer tissues and BALB/c nude mouse model of lung metastasis. The results are summarized in Figure 7a and b. In Figure 7a, IHC analysis was conducted on human lung cancer tissues and adjacent normal lung tissues. The results showed significantly higher expression levels of p-AKT, p-MDM2, and E2F1 in the cancerous tissues compared to the adjacent normal lung tissues. In Figure 7b, we conducted a similar IHC analysis on the BALB/c nude mouse lung metastasis model. The results revealed that the control group exhibited higher expression levels of p-AKT, p-MDM2, and E2F1 in lung metastatic tumors compared to the NCAPD2 knockdown groups. These findings demonstrate that the suppression of NCAPD2 led to decreased activation of the AKT/MDM2/E2F1 pathway in the BALB/c nude mouse metastasis model, which may imply a reduced capacity for metastatic progression.

In summary, NCAPD2 activates the PI3K/AKT pathway, promotes MDM2 phosphorylation and nuclear translocation, reduces E2F1 ubiquitination, and prolongs E2F1 stability. In turn, E2F1 upregulates NCAPD2 transcription, forming a self-reinforcing positive feedback loop that drives LUAD proliferation and metastasis.

## Discussion

In this study, we confirmed the oncogenic role of NCAPD2 in LUAD using both in vitro and in vivo models. NCAPD2 overexpression enhanced proliferation, invasion, and metastasis, whereas silencing NCAPD2 exerted the opposite effect. Mechanistically, NCAPD2 activated the PI3K/Akt pathway, leading to the phosphorylation and nuclear translocation of MDM2. In the nucleus, MDM2 associates with E2F1 and reduces its ubiquitination, thereby stabilizing E2F1; in turn, E2F1 transcriptionally upregulates NCAPD2, forming a positive feedback loop that drives LUAD progression.

### Mechanistic insights

Although condensin complexes are classically recognized for their roles in chromosome architecture, emerging evidence implicates condensin subunits in oncogenic signaling. Our data link the condensin I subunit NCAPD2 to PI3K/AKT signaling, thereby connecting chromatin structural proteins with canonical cancer pathways. Specifically, NCAPD2 activates AKT, which promotes MDM2 phosphorylation and nuclear localization. Once in the nucleus, MDM2 binds to E2F1 and attenuates its ubiquitination, stabilizing E2F1 protein levels. This observation is consistent with earlier work showing that MDM2 can stabilize E2F1 by inhibiting its ubiquitination^8^ and with studies indicating that post-translational modifications and subcellular localization modulate MDM2's control of E2F1 stability.^6^ Moreover, AKT-mediated regulation of MDM2 provides a mechanistic bridge between upstream growth signals and both p53-dependent and p53-independent pathways.^9^^,^^10^ Importantly, our data extend this model by showing that stabilized E2F1 directly enhances NCAPD2 transcription, thereby establishing a self-reinforcing NCAPD2-AKT/MDM2-E2F1 loop. Notably, we do not claim that E2F1 is a direct ubiquitination substrate of MDM2 in our LUAD context; rather, our data support MDM2-dependent stabilization of E2F1. To directly interrogate “substrate” relationships, MDM2 knockdown combined with E2F1 ubiquitination assays will be prioritized in follow-up experiments.

Beyond our NCAPD2-AKT/MDM2-E2F1 model, it is important to place E2F1 in the broader context of LUAD progression. E2F1 is a multifunctional transcription factor implicated in proliferation, apoptosis, and metastasis.^11^^,^^12^ It has been linked to LUAD progression through diverse mechanisms, such as regulation by MCTS1,^13^ FTO-mediated m6A demethylation,^14^ and WNT5A signaling in brain metastasis.^15^ Previous studies also suggested cross-talk between E2F1 and condensin subunits in other cancers.^16^^,^^17^ Our findings reinforce this connection by showing that E2F1 transcriptionally drives NCAPD2, thereby integrating condensin activity with oncogenic signaling. Together, these results identify NCAPD2 as a central node within the AKT/MDM2/E2F1 regulatory network.

Studies specifically investigating NCAPD2 in LUAD remain limited. Consistent with Li et al., ^18^ who reported that NCAPD2 as a potential diagnostic and prognostic marker correlated with clinical stage, our study not only confirms the oncogenic function of NCAPD2 in LUAD but also delineates its upstream–downstream connections and the feedback mechanism by which NCAPD2 amplifies oncogenic signaling through the AKT/MDM2/E2F1 axis. Taken together, these findings not only validate NCAPD2 as an oncogenic driver in LUAD but also position it as a promising therapeutic target within a novel feedback regulatory loop.

### Therapeutic implications

From a translational perspective, NCAPD2 represents a potentially druggable target in LUAD. Although its role as a chromatin-associated subunit of the condensin I complex has traditionally made it challenging to target directly,^19^ recent advances now allow pharmacological intervention of such intracellular proteins. Strategies include small interfering RNAs (siRNAs), antisense oligonucleotides, and proteolysis-targeting chimeras (PROTACs). For example, PROTACs against BRD4 and STAT3 have demonstrated potent antitumor activity,^20^^,^^21^raising the possibility that NCAPD2 could also be amenable to this approach. In addition, nanoparticle-based siRNA delivery systems have shown efficacy in preclinical lung cancer models,^22^ providing another potential route for NCAPD2 inhibition. In line with these findings, other condensin subunits have also been proposed as therapeutic targets in cancer,^23^ further supporting the rationale for targeting NCAPD2 within the condensin family.

Clinically, NCAPD2 inhibition may complement current precision-oncology strategies by converging on the PI3K/AKT axis. For instance, resistance to EGFR-TKIs in LUAD is frequently mediated by bypass activation of PI3K/AKT signaling,^24^ and NCAPD2 inhibition is expected to attenuate this mechanism and restore sensitivity. Similarly, aberrant PI3K/AKT activation promotes immune evasion by reducing T-cell infiltration^25^; thus, NCAPD2 targeting may enhance responses to immune checkpoint blockade. In addition, condensin complex dysregulation has been associated with chemotherapy resistance,^26^ raising the possibility that NCAPD2 inhibition might resensitize tumors to conventional regimens. Taken together, these molecular and clinical considerations position NCAPD2 as a promising target for future therapeutic development in LUAD.

### Limitations and future directions

This study has several limitations. First, although we validated NCAPD2 function in multiple LUAD cell lines and xenograft models, we did not include patient-derived xenografts (PDXs) or organoid models, which better capture tumor heterogeneity. Future studies should incorporate these clinically relevant models to strengthen their translational value. Second, although we propose NCAPD2 as a potential therapeutic target, we did not perform in vivo intervention experiments using pharmacological approaches such as small molecules, siRNA nanoparticles, or PROTACs. Therefore, the translational relevance of NCAPD2 targeting remains to be further tested. Third, our tissue cohort was derived from a single center. Independent validation in multicenter cohorts and with larger sample sizes will be required to confirm the generalizability of our findings.

## Conclusion

In conclusion, our study reveals that NCAPD2 drives LUAD progression through an AKT/MDM2/E2F1 positive feedback loop. This discovery not only clarifies the mechanistic basis of NCAPD2 upregulation in LUAD but also underscores its translational potential as a therapeutic target. Further preclinical and clinical studies are warranted to test the efficacy and safety of NCAPD2-targeted strategies in lung cancer management.

## Methods

### Biological information analysis

This study conducted bioinformatics analysis of LUAD patients from The Cancer Genome Atlas (TCGA). TIMER (cistrome.shinyapps.io/timer) and UALCAN (ualcan.path.uab.edu) are information repositories that integrate TCGA data. The mRNA expression levels of condensin subunits were analyzed across multiple cancer types using the TIMER. Protein expression variations among these subunits were investigated via UALCAN. To evaluate the prognostic impact, the expression levels of these subunits were correlated with survival outcomes. Univariate and multivariate Cox regression analyses were conducted to identify independent risk factors.

The NCAPD2 promoter sequence information was retrieved from the UCSC database (http://genome.ucsc.edu/). Subsequently, an analysis of the potential transcription factors that may bind to the NCAPD2 promoter sequence was conducted using three online resources, namely, TFDB (http://bioinfo.life.hust.edu.cn/AnimalTFDB/),^27^ PROMO (http://alggen.lsi.upc.es/cgi-bin/promo_v3/promo/promoinit.cgi?dirDB=TF_8.3/) and GeneCards (http://www.genecards.org/).

### Patients and tissue samples

Paraffin-embedded tissues were collected from 207 patients diagnosed with LUAD at Fuzhou University Affiliated Provincial Hospital between January 2015 and December 2017. The cohort consisted of 111 female patients and 96 male patients, with ages ranging from 40 to 86 y, and a median age of 67 y. Immunohistochemical analyses were performed on all tissue samples to assess the protein expression of NCAPD2 (ab 198019, 1:100, Abcam), p-AKT (ab 8933, 1:200, Abcam), p-MDM2 (ab 170880, 1:50, Abcam), and E2F1 (ab 94888, 1:100, Abcam). Comprehensive clinical and pathological data were available for all patients. Before analysis, the sample data were anonymized, and the IHC slides were blindly evaluated. The IHC scoring method was performed as previously reported.^28^ Additionally, eight pairs of fresh LUAD and adjacent normal tissues were procured from Fuzhou University Affiliated Provincial Hospital. Written informed consent was obtained from all participants involved in the study. The research was approved by the Ethics Committee of Fuzhou University Affiliated Provincial Hospital (K2020-09-028).

### Cell culture and infection

The human non-small cell lung cancer (NSCLC) cell lines A549, PC9, HCC827, H1975, and H1299, along with the immortalized alveolar epithelial cell line BEAS-2B, were obtained from the Cell Bank of the Chinese Academy of Sciences. All the cell lines underwent authentication via short tandem repeat (STR) profiling to confirm their identity. Authentication procedures were conducted by Procell Life Science & Technology Co., Ltd., following established protocols. The cell lines utilized in this study were most recently authenticated on February 15, 2022. We affirm that the cell lines employed in our research were sourced appropriately, authenticated, and routinely monitored to ensure their identity and integrity throughout the study.

The cells were cultured in their respective recommended media supplemented with 10% fetal bovine serum and maintained at 37 °C with 5% CO_2_. Cycloheximide (CHX; Selleck, Cat# S7418) was used at a concentration of 10 μg/ml.

HCC827, PC9, and A549 cells were each subjected to both overexpression and knockdown of NCAPD2. Overexpression was achieved by transducing cells with lentiviruses, while gene knockdown was performed using short hairpin RNAs (shRNAs) synthesized by Hanheng Biology Co., Ltd. (Shanghai, China). The specific target sequences of the two shRNAs were as follows: sh-NCAPD2-1# (5ʹ- GGTACTGTCCATCAAACATCT -3ʹ) and sh-NCAPD2-2# (5ʹ- GGTTCTCAGTGGCGATCAACT -3ʹ), while the sh-NC (negative control) sequence was 5ʹ- CCGGCAACAAGATGAAGAGCACCAACTCGAGTTGGTGCTCTTCATCTTGTTGTTTTTG -3ʹ.

The overexpression plasmids for HNF1A, SP1, E2F1, YY1, GATA2, and MAZ were purchased from Jikai Company (Shanghai, China). The siRNA sequences targeting E2F1 were as follows: si-E2F1-1# (5ʹ-GGACCACCTGATGAATATCTGTACT-3ʹ) and si-E2F1-2# (5ʹ-GACGTGTCAGGACCTTCGTAGCATT-3ʹ), with the si-NC (negative control) sequence being 5ʹ-UUCUCCGAACGUGUCACGUTT-3ʹ.

### Quantitative reverse transcription PCR (qRT-PCR) analysis

Total RNA was extracted using Trizol reagent (Thermo Fisher Scientific, Cat# 15596026), followed by reverse transcription to cDNA. qPCR was performed using SYBR Green master mix (Thermo Fisher Scientific, Cat# 4309155), and mRNA expression was determined using the 2^−^​​​​​​^△△Ct^ method.

The amplification primers are listed in Table S1.

### Western blotting

The cells were lysed, and protein was extracted and assessed using a BCA protein detection kit (Beyotime Biotechnology, Cat# P0012). Next, 10 µg of protein was separated by SDS‒PAGE, transferred to a PVDF membrane (Millipore, Cat# IPVH00010), and blocked with TBST. The membrane was incubated with primary and secondary antibodies. Protein visualization was performed using a chemiluminescent substrate kit (Thermo Fisher Scientific, Cat# 34580), and the signal was captured with an imaging system. The specific antibodies used are listed in Table S2.

### Cell viability measurement, wound healing assay, transwell assay, and cell cycle analysis

Cell viability was assessed using Cell Counting Kit 8 (CCK-8) and colony formation assays. Cell migration and invasion ability were analyzed through Transwell assays and wound healing assays. Cell cycle analysis was performed using propidium iodide staining. The detailed procedures are provided in Data S1.

### Cell transcriptome sequencing and enrichment analysis

Transcriptional profiling of PC9 cells in the control group and knockdown group was conducted using RNA sequencing. The sequencing process was carried out with assistance from Jikai Company, Shanghai. We analyzed the differential expression fold changes of all genes using the DESeq2 R package, sorting them in descending order based on these changes. To further investigate the differential expression between the control group and the knockdown group, we utilized the gene set enrichment analysis (GSEA) R package to analyze the associated signaling pathways.

### Chromatin immunoprecipitation assay (ChIP)

The Pierce Magnetic ChIP Kit (Thermo Fisher Scientific, Cat# 26157) was used to perform the ChIP assay. A crosslinking step was conducted using a 1% formaldehyde and 1% glycine solution to fix 1 × 10^7^ cells. Subsequently, cell lysis was carried out using the membrane extraction buffer, followed by DNA fragmentation induced by MNase treatment and sonication. The successful fragmentation of the DNA was confirmed by agarose electrophoresis. The resulting DNA fragments were subjected to an overnight incubation with antibodies against RNA polymerase II, IgG, and E2F1. Protein A/G magnetic beads and elution buffers were utilized for the purification of the antibody‒DNA fragment complexes. The enriched DNA fragments were then analyzed for specific enrichment using qRT-PCR. The anti-E2F1 antibody (Cell Signaling Technology, Cat# 3742) was used.

### Dual-luciferase reporter (DR) assay

The NCAPD2 promoter fragment was amplified and inserted into the luciferase reporter vector pGL3, creating pGL3-2000. A mutant version, pGL3-mut, was generated via site-directed mutagenesis. Firefly luciferase and Renilla luciferase signals were measured using the Dual-Luciferase Reporter Assay System (Promega, Cat# E1910) according to the manufacturer's protocol. The experiments were conducted in triplicate for each analysis.

### Immunofluorescence

Cells were seeded in confocal Petri dishes at a density of 5 × 10^4^. After three washes with ice-cold PBS, the cells were fixed with paraformaldehyde. The samples were subsequently permeabilized with 0.1% Triton X-100 and washed again with chilled PBS. To prevent nonspecific binding, the cells were treated with 1% bovine serum albumin (BSA) for 1 h at room temperature. The cells were then incubated overnight at 4 °C with a primary antibody specific to MDM2 (Cell Signaling Technology, Cat# 86934, 1:200). Afterward, a fluorescently labeled secondary antibody (Alexa Fluor® 647, Abcam, Cat# ab150083, 1:1000) was applied to the cells for 1 h in the dark at room temperature. Nuclear staining was performed using DAPI (Beyotime Biotechnology, Cat# C1002, 1:10,000) for 10 min at room temperature, away from light. Finally, the images were acquired and analyzed using a microscope.

### Co-immunoprecipitation (Co-IP)

The human lung adenocarcinoma PC9 cell line was used for this experiment. The cells were lysed in RIPA buffer supplemented with protease inhibitors and incubated on ice for 30 min to ensure complete lysis. The lysates were centrifuged at 12,000 × *g* for 15 min at 4 °C, and the supernatants were collected as input samples for immunoprecipitation. The Co-IP experiment included four groups: the input group (unprocessed cell lysate, directly used for Western blot detection), the IgG negative control group (nonspecific IgG antibody), the E2F1 group (E2F1-specific antibody), and the MDM2 group (MDM2-specific antibody). The respective antibodies were mixed with the cell lysates and incubated at 4 °C for 4 h or overnight. Protein‒antibody complexes were then captured by incubation with protein A/G agarose beads for 1 h at 4 °C. After immunoprecipitation, the beads were washed four times with PBS containing 0.1% Triton X-100 to remove nonspecifically bound proteins. The input group samples and the immunoprecipitated proteins from the other three groups were eluted by boiling in SDS‒PAGE loading buffer for 5 min. Proteins were separated by SDS‒PAGE and transferred to PVDF membranes. The membranes were probed with primary antibodies against E2F1 and MDM2, followed by incubation with horseradish peroxidase (HRP)-conjugated secondary antibodies. Protein bands were visualized using enhanced chemiluminescence.

### Ubiquitination assay

The human lung adenocarcinoma PC9 cell line was used and divided into two groups: the vector control group and the NCAPD2 overexpression group (OE group). Both groups were co-transfected with an HA-ubiquitin (HA-Ub) plasmid. After 24 h of transfection, the cells were treated with 10  µM MG132 for 6 h to inhibit proteasomal degradation. The cells were then lysed in RIPA buffer containing protease inhibitors, and the supernatants were collected as input samples. Western blot analysis was performed to detect the expression levels of E2F1, ubiquitin (Ub), and glyceraldehyde-3-phosphate dehydrogenase (GAPDH), with GAPDH serving as a loading control. The cell lysates were incubated with an E2F1-specific antibody for 4 h, followed by incubation with protein A/G agarose beads to enrich the E2F1-containing complexes. The immunoprecipitated samples were washed four times with PBS containing 0.1% Triton X-100, and the proteins were eluted by boiling in SDS‒PAGE loading buffer for 5 min. The samples were separated by SDS‒PAGE, transferred to PVDF membranes, and probed with an E2F1 antibody to confirm equal levels of E2F1 between the two groups. Ubiquitination of E2F1 was detected using an anti-ubiquitin antibody.

### Tumorigenicity assays in nude mice

All animal experiments were conducted at Fujian University of Traditional Chinese Medicine (FJTCM) under the approval of their Ethics Committee (Approval No: FJTCM IACUC 2021170). Male BALB/c nude mice (6−8 weeks old, weighing 18‒22 g) were obtained from Shanghai Slack Laboratory Animal Co., Ltd. The mice were randomly assigned to three age-matched groups, each containing nine mice, and a double-blind approach was employed for objectivity. LUAD cells with stable NCAPD2 knockdown or negative control were suspended in PBS at a concentration of 1 × 10^7^ cells/mL. 100 μL of this cell suspension was then mixed with an equal volume of Matrigel (BD Biosciences, Cat# 356234), resulting in a final total volume of 200 μL, which was subcutaneously injected into the flanks of BALB/c nude mice. Tumor growth and volume were monitored twice per week. After 28 d, all the mice were euthanized by cervical dislocation under isoflurane anesthesia to ensure a pain-free state, and tumor specimens were collected for further analysis. All procedures were approved by the Institutional Animal Care and Use Committee (IACUC) and conducted in accordance with ethical guidelines to minimize animal suffering.

Tissue specimens were fixed in 10% formalin and embedded in paraffin. H&E staining was performed for histological examination. Immunohistochemical (IHC) staining was conducted using the Ki-67 antibody (Servicebio, Cat# GB111141, 1:500), p-AKT (Abcam, Cat# ab8933, 1:200), p-MDM2 (Abcam, Cat# ab170880, 1:50), and E2F1 antibodies (Abcam, Cat# ab94888, 1:100).

### Tail vein injection model for assessing lung adenocarcinoma metastasis

Six- to eight-week-old male BALB/c nude mice were randomly divided into three groups (*n* = 9/group). 0.1 ml of stable Luc-PC9 cells (containing 1 × 10^6^ cells) transfected with either luciferase-tagged shNCAPD or shNC control were tail-vein injected. Tumor growth was monitored every 14 d using bioluminescence imaging. At week 8, the mice were euthanized by cervical dislocation under isoflurane anesthesia, and the lungs were fixed in 10% formalin. H&E staining was performed to assess lung metastasis. Images were captured under a microscope.

### Statistical analysis

Data analysis was performed using GraphPad Prism 7.0 (GraphPad Software, Inc., San Diego, CA, USA). Each experiment was conducted in triplicate, and the data were presented as median (IQR) for nonnormally distributed data and as mean ± SD for normally distributed data. For comparisons between two independent groups with normal distribution, a two-tailed Student's *t*-test was applied. For comparisons between three or more independent groups with a normal distribution, one-way analysis of variance (ANOVA) was performed, followed by the Bonferroni post hoc test for pairwise comparisons when significant differences were detected. For nonnormally distributed data, the Kruskal‒Wallis test was used to compare differences among three or more groups, followed by Dunn's post hoc test for pairwise comparisons. For all paired data, if the data were normally distributed, paired *t*-tests were applied. For nonnormally distributed paired data, the Wilcoxon rank-test was used for comparison. Kaplan‒Meier survival curves were generated to assess overall survival, and the significance of differences was evaluated using the log-rank test. The analysis was conducted using R software with the survival and survminer packages. A *p*-value of <0.05 was considered statistically significant.

## Consent for publication

The consents for publication was obtained from all participants.

## Consent to participate

Written consent for participation was obtained from all participants.

## Supplementary Material

Supplementary material Figure legend

Supplementary material Table S1

Supplementary material Figure S1

Supplementary material Table S3

Supplementary material Figure_S4.R2

Supplementary material Figure_S5.R2

Supplementary material Data S1

Supplementary material Table S2

Supplementary material Figure_S3.R2

Supplementary material Figure_S2.R2

## Data Availability

The data that support the findings of this study are openly available in The Cancer Genome Atlas (TCGA) data portal (https://tcga-data.nci.nih.gov/tcga/). The raw RNA sequencing data generated in this study have been deposited in the Gene Expression Omnibus (GEO) database under accession number GSE280143 (https://www.ncbi.nlm.nih.gov/geo/query/acc.cgi?acc=GSE280143). The rest of the data are available from the corresponding author upon reasonable request.

## Abbreviations

NSCLC: non-small cell lung cancer
LUAD: lung adenocarcinoma
TCGA: The Cancer Genome Atlas
TIMER: Tumor IMmune Estimation Resource
UALCAN: University of Alabama at Birmingham Cancer data analysis portal
qRT-PCR: quantitative real-time polymerase chain reaction
IHC: immunohistochemistry
H&E: hematoxylin and eosin
EMT: epithelial–mesenchymal transition
PI3K/AKT: phosphatidylinositol 3-kinase/AKT pathway
LY294002: PI3K inhibitor
CCK-8: Cell Counting Kit-8
ChIP: chromatin immunoprecipitation
Co-IP: co-immunoprecipitation
OE: overexpression
shRNA (sh-): short hairpin RNA
TFDB: transcription factor database
GeneCards: genecards database
PROMO: transcription factor binding site prediction tool
JASPAR: nonredundant transcription factor binding motif database
BS: binding site (e.g., BS1/BS2)
EGFR-TKI: epidermal growth factor receptor tyrosine kinase inhibitor
PROTAC: proteolysis-targeting chimera
PDX: patient-derived xenograft
CHX: cycloheximide
