# Supplementary material for: NCAPD2 promotes the progression of lung adenocarcinoma through an AKT/MDM2/E2F1 positive feedback loop
Source: Cancer Biol Ther. 2025 Nov 30;26(1):2589678. doi: 10.1080/15384047.2025.2589678 (PMC12676955; doi:10.1080/15384047.2025.2589678)
Supplement: Supplementary material — Figure legend [file KCBT_A_2589678_SM1146.docx]

**Figure legends**

**Figure 1** Overexpression of NCAPD2 in lung adenocarcinoma and its promotion of proliferation.

a. NCAPD2 was the most significant difference in expression between lung adenocarcinoma tissue and adjacent tissues.

b. Representative immunohistochemical staining images of NCAPD2.

c. Correlation between NCAPD2 expression and pathological staging.

d. Elevated expression of NCAPD2 in lung adenocarcinoma patients compared to normal lung tissues (TCGA database).

e. High NCAPD2 expression correlates with poor survival.

f. NCAPD2 expression increases with advancing clinical stages.

g. Western blot analysis showing increased NCAPD2 expression in LUAD tissues compared to adjacent normal lung tissues from 8 paired samples.

h. Increased NCAPD2 expression in lung adenocarcinoma cell lines (A549, PC9, HCC827, H1975, and H1299) compared to alveolar epithelial cells (BEAS-2B) (Western blotting).

i. Significant knockdown of NCAPD2 with shNCAPD2-1# and shNCAPD2-2# at the protein level, while OE-NCAPD2 exhibits higher protein levels than the control group (Western blotting).

j-k. NCAPD2 overexpression promotes proliferation in HCC827 cells (CCK-8 and clone formation assays), while NCAPD2 knockdown inhibits proliferation in PC9 cells.

* p<0.05, **p<0.01, ***p<0.001

VE: empty vector control, OE: overexpression, sh: short hairpin, TPM: transcripts per million

**Figure 2** Effects of NCAPD2 on cell cycle progression and in vivo tumor growth in lung adenocarcinoma.

a. NCAPD2 alters cell cycle distribution in HCC827 and PC9 cells.

b. Expression of cell cycle-related markers (CCND1, CCNE1, P27) in PC9 and HCC827 cells.

c. Photographs of xenograft tumors.

d-e. The volume and mass of tumors formed by the NCAPD2-knockdown cell lines were significantly smaller compared to the control group.

f-g. H&E staining and immunohistochemical staining of Ki-67 in xenograft tumors.

* p<0.05, **p<0.01, ***p<0.001

(H&E): hematoxylin and eosin

**Figure 3** NCAPD2 promotes migration, invasion, and epithelial-mesenchymal transition (EMT) in lung adenocarcinoma cells.

a. NCAPD2 overexpression enhances HCC827 cell migration (wound healing assay), while NCAPD2 knockdown weakens PC9 cell migration.

b. NCAPD2 overexpression increases HCC827 cell migration and invasion (Transwell assay), while NCAPD2 knockdown decreases migration and invasion in PC9 cells.

c. Expression of EMT-related markers (E-cadherin, N-cadherin, MMP9) in PC9 and HCC827 cells.

d. Assessment of invasive and metastatic capacities via small animal in vivo imaging following NCAPD2 knockdown.

e. Representative H&E images of lung metastases in the mouse model

* p<0.05, **p<0.01, ***p<0.001

**Figure 4** NCAPD2 promotes lung adenocarcinoma progression via the PI3K/AKT signaling pathway.

a. GSEA analysis showed downregulation of PI3K/AKT pathway in NCAPD2 knockdown cells.

b. NCAPD2 promotes lung adenocarcinoma via PI3K/AKT phosphorylation.

c. LY294002 reverses PI3K/AKT activation caused by NCAPD2 overexpression.

d. LY294002 eliminates increased proliferation induced by NCAPD2 overexpression.

e. LY294002 restores heightened invasion in NCAPD2-overexpressing cells.

f. LY294002 abolishes the alteration expression of E-cadherin, N-cadherin, and MMP9 in NCAPD2-overexpressing cells.

LY294002: a PI3K protein kinase inhibitor

* p<0.05, **p<0.01, ***p<0.001

**Figure 5** Transcription factor E2F1 positively regulates NCAPD2 expression in lung adenocarcinoma.

a. HNF1A, SP1, E2F1, YY1, GATA2, and MAZ were identified as potential regulators of NCAPD2 based on binding site analysis.

b. Overexpression efficiencies of HNF1A, SP1, E2F1, YY1, GATA2, and MAZ in HCC827 and PC9 cells, as verified by qRT-PCR.

c. E2F1 exhibited significantly higher transcriptional activity compared to other transcription factors, including HNF1A, SP1, YY1, GATA2, and MAZ, when co-transfected with the pGL3-2000 luciferase reporter plasmid into PC9 and HCC827 cells.

d. E2F1 knockdown and overexpression resulted in significant changes in mRNA expression level.

e. Overexpression of E2F1 increased NCAPD2 expression, while E2F1 knockdown had the opposite effect.

f. JASPAR predicted two potential E2F1 binding sites in the NCAPD2 promoter region.

g. ChIP assay confirmed E2F1 binding to the BS2 site but not the BS1 site.

h. PCR products from ChIP were visualized by gel electrophoresis, supporting specific binding of E2F1 to the BS2 site

i. DR assay showed reduced luciferase activity with the BS2 mutation site.

j. The heatmap depicts the differential expression of key genes in the PI3K/AKT pathway and E2F1 across PC9 cell lines, comparing the control group and NCAPD2 knockdown group.

* p<0.05, **p<0.01, ***p<0.001

DR: double luciferase reporter, ChIP: chromatin immunoprecipitation

**Figure 6** NCAPD2 regulates the progression of lung adenocarcinoma through the AKT/MDM2/E2F1 positive feedback loop.

a-b. NCAPD2 overexpression upregulated E2F1 and p-MDM2 protein levels, while knockdown downregulated them.

c. NCAPD2 overexpression enhanced MDM2 nuclear translocation.

d. MDM2 and E2F1 were found to interact through co-immunoprecipitation.

e. LY294002 treatment reversed the increased expression of E2F1 and p-MDM2 caused by NCAPD2 overexpression.

f. LY294002 prevented the nuclear increase of MDM2 in NCAPD2-overexpressing cells.

g. NCAPD2 overexpression extended the half-life of E2F1 in the presence of CHX.

h. Immunoprecipitation assay showing reduced ubiquitination of E2F1 upon NCAPD2 overexpression.

i. Overexpression of E2F1 enhanced the activation of AKT and MDM2, while knockdown of NCAPD2 significantly reduced the phosphorylation levels of both AKT and MDM2.

* p<0.05, **p<0.01, ***p<0.001

**Figure 7** Immunohistochemical analysis of AKT/MDM2/E2F1 pathway markers in human LUAD tissues and adjacent normal tissues, as well as in BALB/c nude mouse lung metastasis models.

a. The expression levels of p-AKT, p-MDM2, and E2F1 are higher in human lung cancer tissues compared to adjacent normal lung tissues.

b. In the BALB/c nude mouse lung metastasis model, expression of these markers is elevated in the control group (shNC) compared to the NCAPD2 knockdown groups (sh-1# and sh-2#), suggesting the involvement of NCAPD2 in activating the AKT/MDM2/E2F1 signaling pathway.

* p<0.05, **p<0.01, ***p<0.001

**Supplementary Information**

**Table S1** Primer used in this study.

**Table S2** Antibodies used in this study.

**Table S3** Univariate and multivariate Cox regression analysis results highlighting the independent risk factors among condensin subunits in lung adenocarcinoma.

**Data S1** Specific steps for cell viability assay, wound healing assay, Transwell assay, and cell cycle analysis.

**Figure S1** Expression analysis of condensin subunits in lung adenocarcinoma.

**Figure S2** Protein expression differences of condensin subunits in lung adenocarcinoma.

**Figure S3** Correlation between expression levels of condensin subunits and survival in lung adenocarcinoma.

**Figure S4**

a. Overexpression efficiency of NCAPD2 in PC9 cells and the knockdown efficiency of NCAPD2 in HCC827 cells, as determined by qRT-PCR.

b-c. NCAPD2 overexpression promotes proliferation in PC9 cells (CCK-8 and clone formation assays), while NCAPD2 knockdown inhibits proliferation in HCC827 cell.

d-e. NCAPD2 overexpression enhances PC9 cell invasion and migration (wound healing assay and Transwell assay), while NCAPD2 knockdown weakens HCC827 cell invasion and migration.

f. Overexpression and knockdown efficiencies of NCAPD2 in A549 cells, as determined by qRT-PCR.

g-h. NCAPD2 overexpression enhances A549 cell proliferation, invasion, and migration (CCK8 assay and Transwell assay), while NCAPD2 knockdown weakens A549 cell proliferation, invasion, and migration.

* p<0.05, **p<0.01, ***p<0.001

**Figure S5**

a. Western blot analysis of pathway molecules in the rescue experiment. PC9 cells were transfected with shNC or shNCAPD2 and co-transfected with vector (VE) or E2F1-overexpression plasmid (OE-E2F1). NCAPD2 knockdown decreased p-AKT (Ser473), p-MDM2 (Ser166), and E2F1 expression, whereas E2F1 overexpression restored these signals.

b. CCK-8 proliferation assay for the same groups. Cell viability was significantly reduced by NCAPD2 knockdown and partially restored by E2F1 overexpression.

c. Transwell migration and invasion assays in the rescue experiment. Representative images (left) and quantitative analysis (right) show that the reduced migratory and invasive abilities caused by NCAPD2 knockdown were partially rescued by E2F1 overexpression.

d. Western blot analysis of pathway molecules in the requirement experiment. PC9 cells were transfected with si-NC or si-MDM2 together with vector (VE) or NCAPD2-overexpression plasmid (OE-NCAPD2). NCAPD2 overexpression increased p-AKT (Ser473), p-MDM2 (Ser166), and E2F1 expression, while MDM2 knockdown abolished the increases in p-MDM2 and E2F1 but not p-AKT.

e. CCK-8 proliferation assay for the requirement experiment. NCAPD2 overexpression enhanced cell proliferation, whereas MDM2 knockdown abolished this effect.

f. Transwell migration and invasion assays for the requirement experiment. NCAPD2 overexpression markedly increased cell migration and invasion, which were abolished by MDM2 knockdown.

* p<0.05, **p<0.01, ***p<0.001
