# Supplementary material for: NCAPD2 promotes the progression of lung adenocarcinoma through an AKT/MDM2/E2F1 positive feedback loop
Source: Cancer Biol Ther. 2025 Nov 30;26(1):2589678. doi: 10.1080/15384047.2025.2589678 (PMC12676955; doi:10.1080/15384047.2025.2589678)
Supplement: Supplementary material — Table S1 [file KCBT_A_2589678_SM1145.docx]

**Table S1** Primer used in this study.

|  | Sequence (5’ to 3’) |
| --- | --- |
| SMC4-Forward | ctggagctcctcggcttatg |
| SMC4-Reverse | catcgtgttcagtctggcct |
| NCAPG-Forward | gcccctgcatcttctccttt |
| NCAPG-Reverse | cagcagtctgacacctcctg |
| NCAPH-Forward | acgatgaacctgaccacacc |
| NCAPH-Reverse | tcatcactgtcagcagcctg |
| NCAPD2-Forward | gacctggcctactgtgtgtc |
| NCAPD2-Reverse | ctaggatctgtgcctgcgag |
| NCAPG2-Forward | aaatgatgcccccgaccatt |
| NCAPG2-Reverse | cctctggaggctctctcact |
| E2F1-Forward | gcatccagctcattgccaag |
| E2F1-Reverse | gttcttgctccaggctgagt |
| HNF1A-Forward | agacgctagtggaggagtgcaa |
| HNF1A-Reverse | ggcaaaccagttgtagacacgc |
| SP1-Forward | acgcttcacacgttcggatgag |
| SP1-Reverse | tgacaggtggtcactcctcatg |
| YY1-Forward | ggaggaatacctggcattgacc |
| YY1-Reverse | ccctgaacatctttgtgcagcc |
| GATA2-Forward | cagcaaggctcgttcctgttca |
| GATA2-Reverse | atgagtggtcggttctgcccat |
| MAZ-Forward | ggatcacctcaacagtcacgtc |
| MAZ-Reverse | ggcactttctcctcgtgtcgta |
| GAPDH- Forward | acaactttggtatcgtggaagg |
| GAPDH- Reverse | gccatcacgccacagtttc |
