# Supplementary figures and images for: NCAPD2 promotes the progression of lung adenocarcinoma through an AKT/MDM2/E2F1 positive feedback loop
Source: Cancer Biol Ther. 2025 Nov 30;26(1):2589678. doi: 10.1080/15384047.2025.2589678 (PMC12676955; doi:10.1080/15384047.2025.2589678)

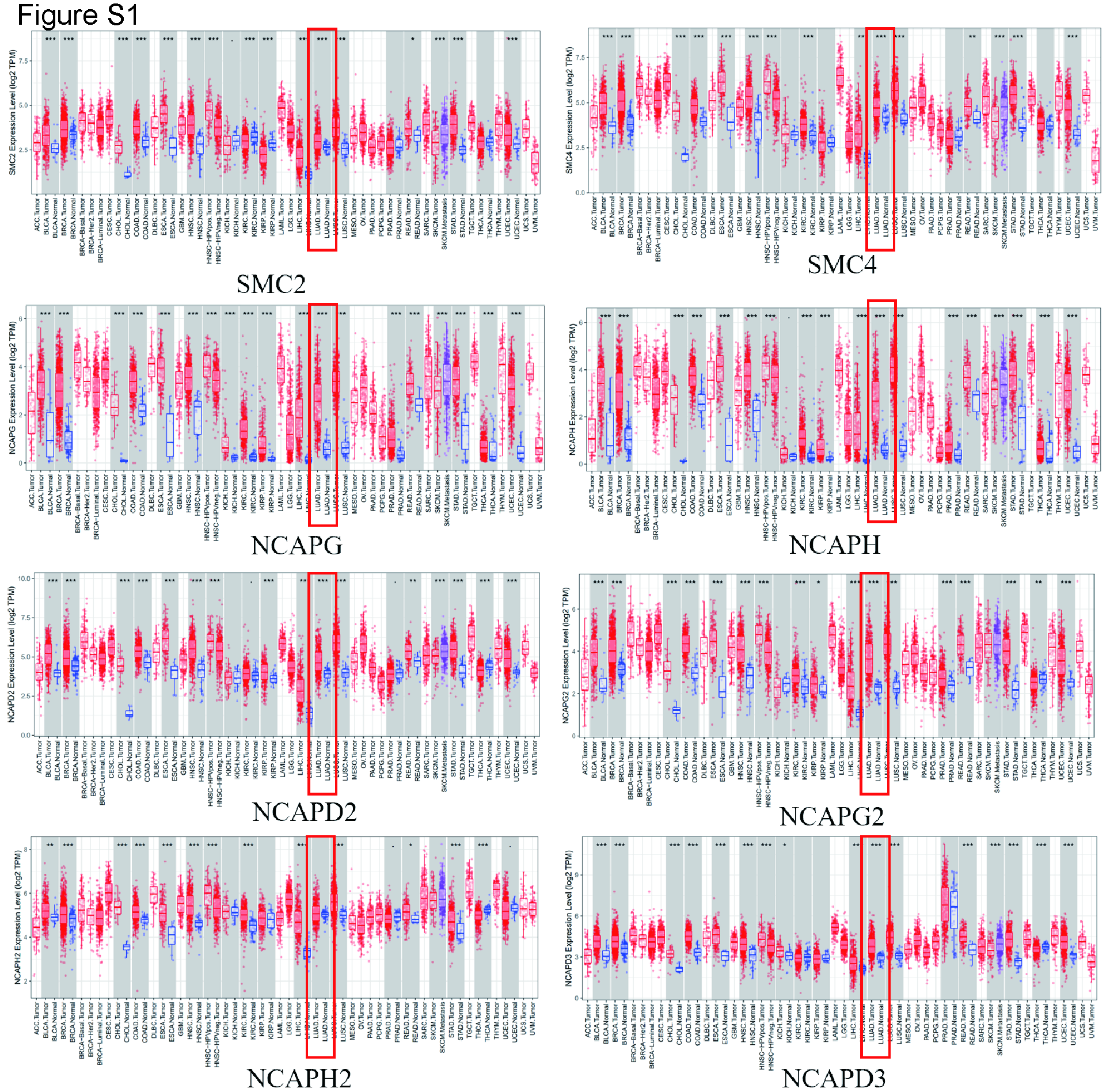

Supplement: Supplementary material — Figure S1 [file KCBT_A_2589678_SM1144.tif]

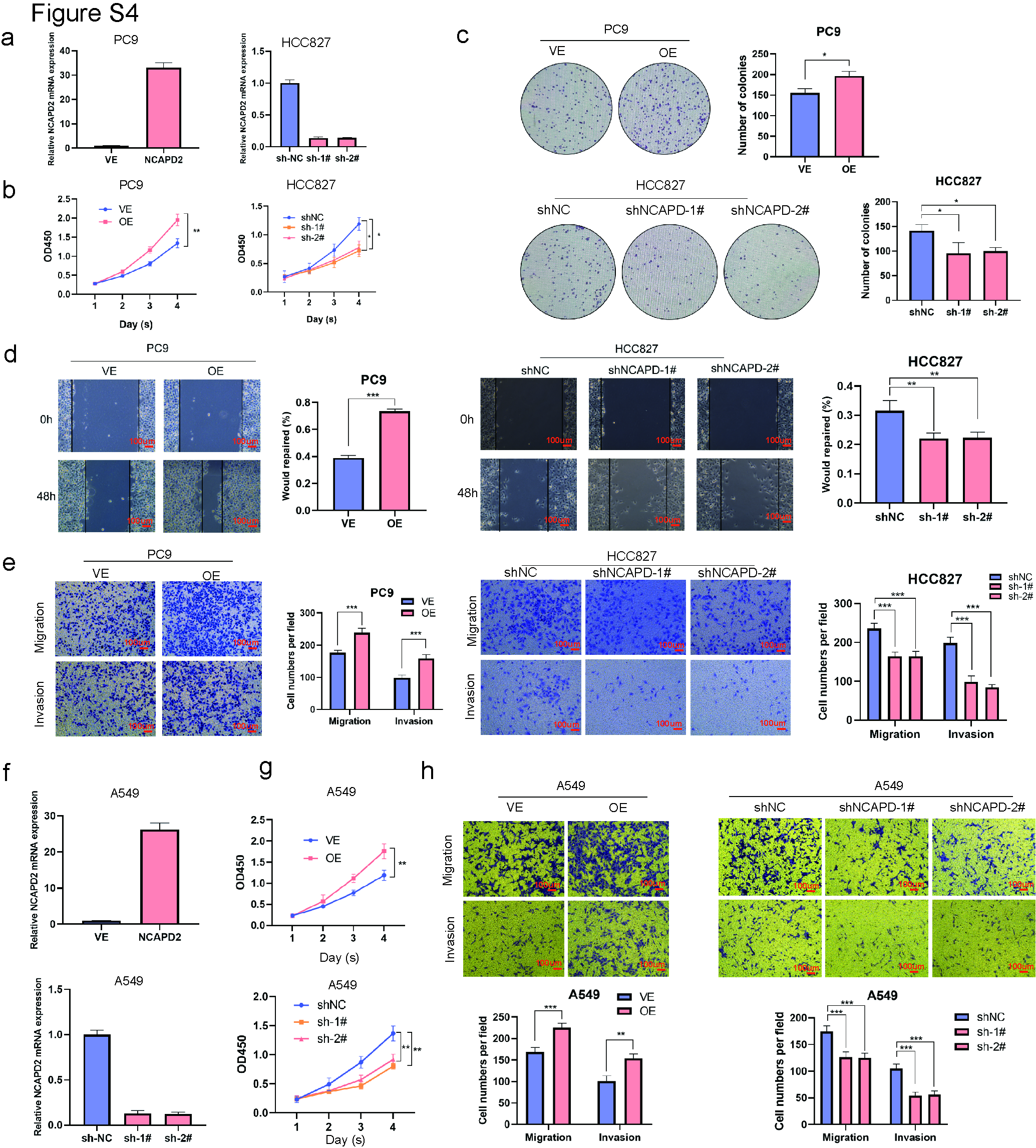

Supplement: Supplementary material — Figure_S4.R2 [file KCBT_A_2589678_SM1142.tif]

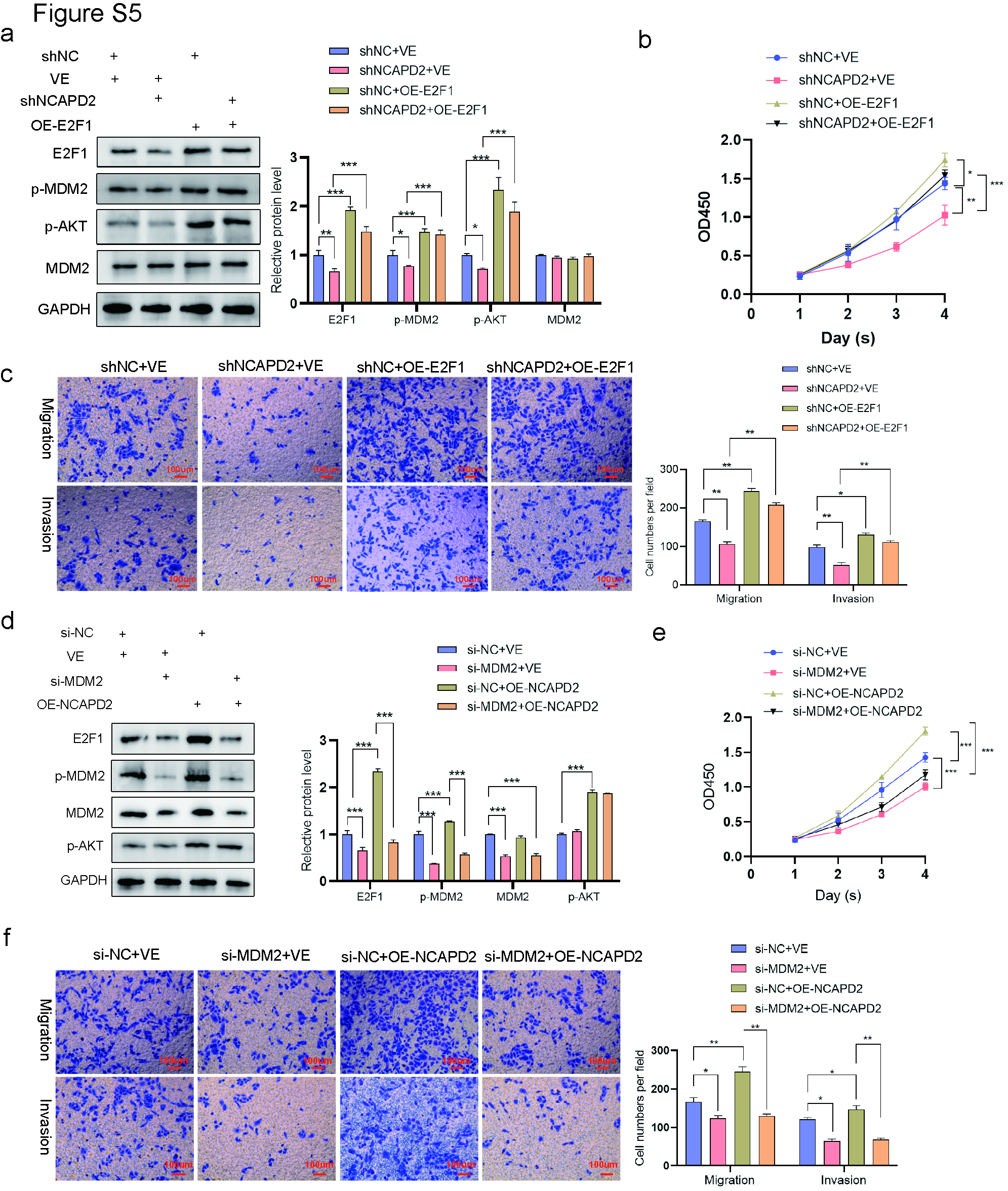

Supplement: Supplementary material — Figure_S5.R2 [file KCBT_A_2589678_SM1141.tif]

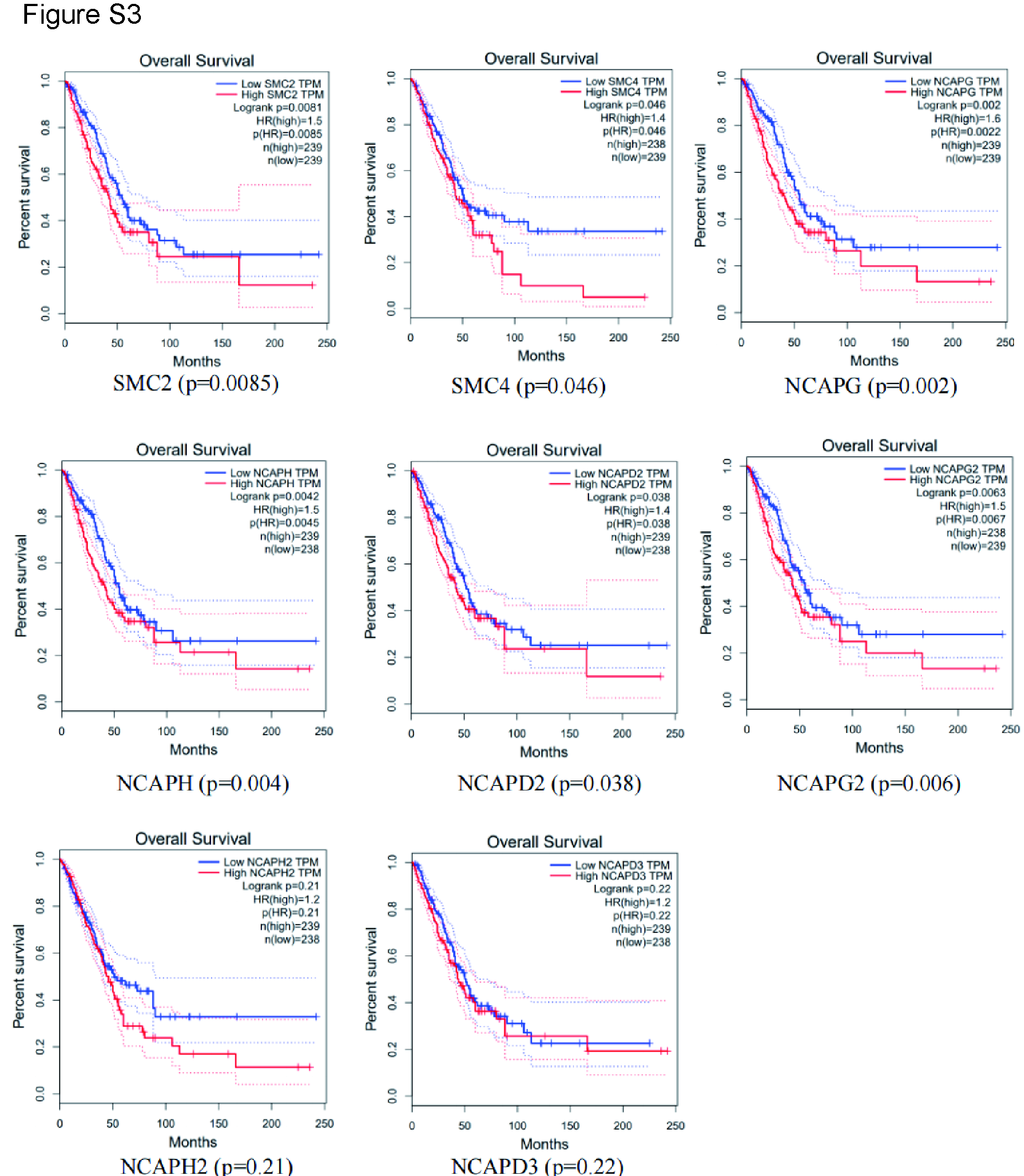

Supplement: Supplementary material — Figure_S3.R2 [file KCBT_A_2589678_SM1138.tif]

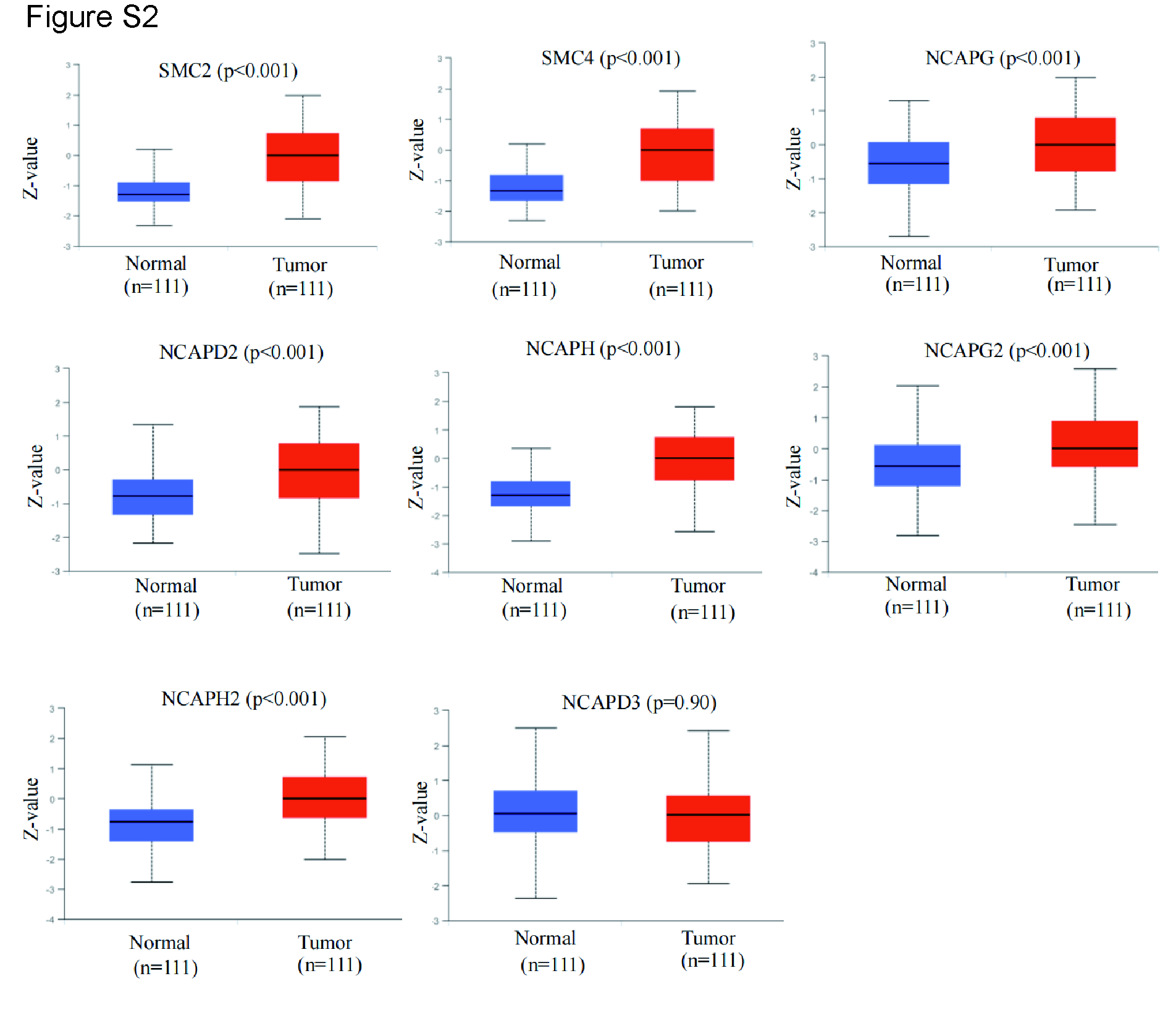

Supplement: Supplementary material — Figure_S2.R2 [file KCBT_A_2589678_SM1137.tif]
