# Supplementary material for: NCAPD2 promotes the progression of lung adenocarcinoma through an AKT/MDM2/E2F1 positive feedback loop
Source: Cancer Biol Ther. 2025 Nov 30;26(1):2589678. doi: 10.1080/15384047.2025.2589678 (PMC12676955; doi:10.1080/15384047.2025.2589678)
Supplement: Supplementary material — Table S3 [file KCBT_A_2589678_SM1143.docx]

**Table S3** Univariate and multivariate Cox regression analysis results highlighting the independent risk factors among condensin subunits in lung adenocarcinoma.

| Gene | HR(95% CI)  Univariate analysis | P value | HR(95% CI)  Multivariate analysis | P value |
| --- | --- | --- | --- | --- |
| SMC2 | 1.391 (1.125-1.720) | 0.002 | 1.586 (1.242-2.027) | <0.001 |
| SMC4 | 1.110 (0.932-1.321) | 0.243 |  |  |
| NCAPG | 1.285 (1.113-1.485) | <0.001 | 1.335 (1.125-1.584) | <0.001 |
| NCAPH | 1.240 (1.078-1.426) | 0.003 | 1.248 (1.057-1.473) | 0.009 |
| NCAPD2 | 1.348 (1.132-1.606) | <0.001 | 1.294 (1.047-1.598) | 0.017 |
| NCAPG2 | 1.230 (1.029-1.470) | 0.023 | 1.299 (1.049-1.608) | 0.016 |
| NCAPH2 | 0.955 (0.754-1.210) | 0.703 |  |  |
| NCAPD3 | 1.094 (0.875-1.369) | 0.431 |  |  |
