# Supplementary material for: NCAPD2 promotes the progression of lung adenocarcinoma through an AKT/MDM2/E2F1 positive feedback loop
Source: Cancer Biol Ther. 2025 Nov 30;26(1):2589678. doi: 10.1080/15384047.2025.2589678 (PMC12676955; doi:10.1080/15384047.2025.2589678)
Supplement: Supplementary material — Data S1 [file KCBT_A_2589678_SM1140.docx]

***Proliferation assays***

We assessed the proliferation capacity of HCC827 and A549 cells using the CCK8 assay and colony formation assay.

For the CCK8 assay, cells were infected with lentivirus and then treated with CCK8 reagent (Beyotime Biotechnology, Shanghai, China). The absorbance at 450 nm was measured at 24, 48, 72, and 96 hours after treatment.
In the colony formation assay, 1000 cells were seeded in each well of a six-well plate. After 7 days of incubation, the colonies were fixed, stained, and the number of formed colonies was quantified.

***Migration assays***

We assessed the migration of HCC827 and A549 cells using the wound-healing and transwell assays, respectively.

For the wound healing assay, cells were cultured in a six-well plate at a density of 5×10^5^ cells/well. Once the cells reached 90% confluence, a straight line was vertically drawn at the bottom of each well using a 10 μL pipette tip. Debris was carefully removed, and the cells were cultured in serum-free RPMI-1640 medium. Photographic images of the wound were captured at 0 and 48 hours to evaluate cell migration.

In the transwell migration assay, 200 μL of cell suspension (1×10^5^/mL) in serum-free RPMI-1640 medium was added to the upper chamber, while 600 μL of medium containing 20% fetal bovine serum without cells was added to the lower chamber. After 24 hours, the upper chamber was gently removed, washed, fixed with paraformaldehyde, and stained with crystal violet. Non-migrating cells in the upper chamber were wiped off using a cotton swab, and photographs were taken. Five randomly selected fields of view were used for cell counting. For the invasion experiment, the upper chamber was pre-coated with Matrigel (BD Biosciences, San Jose, CA) before adding the cells. The subsequent steps were similar to those of the migration experiments.

***Cell cycle analysis***

Cell cycle analysis utilized a cell cycle kit (cat. no. C1052; Beyotime Institute of Biotechnology, Haimen, China). After washing the cells with cold PBS, they were fixed in ice-cold 70% ethanol (-20°C) for 12 hours. Subsequently, the cells were resuspended in PBS containing ribonuclease (cat. no. C1052-3; Beyotime Institute of Biotechnology) and stained with propidium iodide (PI) staining buffer. The staining was conducted at 37°C for 30 minutes in the dark. Flow cytometry analysis was performed using a BD FACSVerse flow cytometer (BD Biosciences, Franklin Lakes, NJ, USA), and the distribution of cells in the G0/G1, S, and G2/M phases was determined using FlowJo software. The experiments were repeated three times to ensure statistical robustness.
