# Supplementary material for: NCAPD2 promotes the progression of lung adenocarcinoma through an AKT/MDM2/E2F1 positive feedback loop
Source: Cancer Biol Ther. 2025 Nov 30;26(1):2589678. doi: 10.1080/15384047.2025.2589678 (PMC12676955; doi:10.1080/15384047.2025.2589678)
Supplement: Supplementary material — Table S2 [file KCBT_A_2589678_SM1139.docx]

**Table S2** Antibodies used in this study

| Protein | Cat no. | Company | Source | KD |
| --- | --- | --- | --- | --- |
| NCAPD2 | ab137075 | Abcam | Rabbit | 157 |
| E-cadherin | #14472 | CST | Mouse | 135 |
| N-cadherin | #13116 | CST | Rabbit | 140 |
| MMP9 | #13667 | CST | Rabbit | 84 |
| Cyclin D1 | #55506 | CST | Rabbit | 36 |
| Cyclin E1 | #20808 | CST | Rabbit | 48 |
| P27 | #3686 | CST | Rabbit | 27 |
| MDM2 | #86934 | CST | Rabbit | 90 |
| Phospho-MDM2 (Ser166) | #3521 | CST | Rabbit | 90 |
| Akt | #4685 | CST | Rabbit | 60 |
| Phospho-Akt (Ser 473) | #9271 | CST | Rabbit | 60 |
| Phospho-GSK3β(S9) | ab75814 | Abcam | Rabbit | 46 |
| GSK3β | ab32391 | Abcam | Rabbit | 46 |
| Phospho-mTOR(S2448) | ab109268 | Abcam | Rabbit | 289 |
| mTOR | ab134903 | Abcam | Rabbit | 289 |
| GAPDH | 51332 | CST | Mouse | 37 |
